# An N-Terminal Missense Mutation in *STX11* Causative of FHL4 Abrogates Syntaxin-11 Binding to Munc18-2

**DOI:** 10.3389/fimmu.2013.00515

**Published:** 2014-01-14

**Authors:** Martha-Lena Müller, Samuel C. C. Chiang, Marie Meeths, Bianca Tesi, Miriam Entesarian, Daniel Nilsson, Stephanie M. Wood, Magnus Nordenskjöld, Jan-Inge Henter, Ahmed Naqvi, Yenan T. Bryceson

**Affiliations:** ^1^Centre for Infectious Medicine, Department of Medicine, Karolinska Institutet, Karolinska University Hospital Huddinge, Stockholm, Sweden; ^2^Childhood Cancer Research Unit, Department of Women’s and Children’s Health, Karolinska Institutet, Karolinska University Hospital Solna, Stockholm, Sweden; ^3^Clinical Genetics Unit, Department of Molecular Medicine and Surgery, Karolinska Institutet, Karolinska University Hospital Solna, Stockholm, Sweden; ^4^Science for Life Laboratory, Department of Molecular Medicine and Surgery, Karolinska Institutet, Stockholm, Sweden; ^5^Division of Hematology and Oncology, Hospital for Sick Children, Toronto, ON, Canada; ^6^Broegelmann Research Laboratory, Institute of Clinical Sciences, University of Bergen, Bergen, Norway

**Keywords:** familial hemophagocytic lymphohistiocytosis, syntaxin-11, Munc18-2, N-peptide

## Abstract

Familial hemophagocytic lymphohistiocytosis (FHL) is an often-fatal hyperinflammatory disorder caused by autosomal recessive mutations in *PRF1, UNC13D, STX11*, and *STXBP2*. We identified a homozygous *STX11* mutation, c.173T > C (p.L58P), in three patients presenting clinically with hemophagocytic lymphohistiocytosis from unrelated Pakistani families. The mutation yields an amino acid substitution in the N-terminal Habc domain of syntaxin-11 and resulted in defective natural killer cell degranulation. Notably, syntaxin-11 expression was decreased in patient cells. However, in an ectopic expression system, syntaxin-11 L58P was expressed at levels comparable to wild-type syntaxin-11, but did not bind Munc18-2. Moreover, another N-terminal syntaxin-11 mutant, R4A, also did not bind Munc18-2. Thus, we have identified a novel missense *STX11* mutation causative of FHL type 4. The syntaxin-11 R4A and L58P mutations reveal that both the N-terminus and Habc domain of syntaxin-11 are required for binding to Munc18-2, implying similarity to the dynamic binary binding of neuronal syntaxin-1 to Munc18-1.

## Introduction

Hemophagocytic lymphohistiocytosis (HLH) is a hyperinflammatory disorder clinically diagnosed based on fulfillment of five out of eight criteria including fever, splenomegaly, bicytopenia, hypertriglyceridemia and/or hypofibrinogenemia, hemophagocytosis, low/absent natural killer (NK) cell activity, hyperferritinemia, and high soluble interleukin (IL)-2 receptor levels (1). Familial hemophagocytic lymphohistiocytosis (FHL) typically presents in infancy (2, 3). The incidence of FHL has been estimated to 1 in 50,000 live births (4). Chemo- and immunotherapy succeeds in controlling the disease in the majority of patients, but persistent remission is rarely obtained. At present, hematopoietic stem cell transplantation (HSCT) is the only cure for FHL (5).

Familial hemophagocytic lymphohistiocytosis is associated with autosomal recessive mutations in genes including *PRF1, UNC13D, STX11*, and *STXBP2* (6–10). In addition, Griscelli syndrome type 2 and Chediak Higashi syndrome, associated with autosomal recessive mutations *RAB27A* and *LYST*, respectively, may also present with HLH and are in addition characterized by hypopigmentation. These genes encode proteins required for cytotoxic granule biogenesis, secretion, and target cell death (11). *STX11*, associated with FHL type 4 (FHL4), has the shortest coding sequence among these genes and accounts for only a small fraction of FHL patients. Relative to other FHL subtypes, patients with *STX11* non-sense mutations or *Stx11*-deficient mice typically display less severe disease (12, 13). Although syntaxin-11 (Stx11)-deficiency abrogates degranulation by both cytotoxic T cells and NK cells (14, 15). The exact molecular mechanisms are not clear. Stx11 has been shown to bind Munc18-2, as well as the SNARE domain-containing proteins SNAP-23 and Vti1b (9, 10, 16, 17). Missense mutations can be informative in elucidating how Stx11 acts to facilitate exocytosis. To date, only two *STX11* missense mutations have been reported (18).

In this study, we report a novel *STX11* missense mutation in three unrelated Pakistani families. The autosomal recessive mutation abrogated NK cell degranulation. Interestingly, biochemical analyses of this N-terminal mutation, in addition to another mutation at the conserved N-terminus of Stx11, revealed binding of the N-terminal Habc domain of Stx11 to Munc18-2, stabilizing Stx11 expression, and facilitating cytotoxic lymphocyte exocytosis.

## Materials and Methods

### Patients and controls

The studies were approved by the ethics committee at the Karolinska Institutet. Written consent was obtained from the patients’ families.

### Cells and antibodies

Peripheral blood mononuclear cells (PBMC) were isolated from peripheral blood by density gradient centrifugation (Lymphoprep, Axis-Shield) and maintained in complete medium (RPMI 1640 supplemented with 10% FBS and 2 mM l-glutamine; all Invitrogen). LAK cells were generated as previously described (19). The human erythroleukemia K562 and mouse mastocytoma P815 cell lines were maintained in complete medium. HEK-293T cells were maintained in DMEM (Invitrogen) supplemented with 10% FBS. Rabbit polyclonal anti-Stx11 and Munc18-2 (Proteintech Group) as well as mouse monoclonal anti-HA (clone 16B12, Covance) and anti-actin (C4, Fischer Scientific) antibodies were used for Western blotting. Mouse monoclonal anti-FLAG (M2, Sigma) was used for immunoprecipitation.

### Functional assays

For assessment of NK cell-mediated cytotoxicity, a standard 4-h ^51^Cr assay was used (14). Cytotoxic lymphocyte exocytosis was assessed by flow cytometry, as previously described (15). Samples were acquired on a Calibur instrument (BD Biosciences) and analyzed using Flowjo 9.4 software (Tree Star).

### Plasmids and sequence analyses

Sequences encoding human Stx11 and Munc18-2 were cloned into a pDisplay vector backbone (Invitrogen) for expression on N-terminally tagged proteins. Stx11 mutations were generated by site-directed mutagenesis. Sequence analyses, alignments, and phylogenetic trees were performed and created with CLC Main Workbench software (v.6).

### Biochemical analyses

Patient and control PBMC or LAK cells were lysed in lysis buffer [20 mM Tris, pH 7.4, 2 mM EDTA, 1% Triton-X-100, 10% glycerol, 100 mM NaCl, protease inhibitors (Roche)]. The protein concentration in nuclei-depleted lysates was determined using Bradford assay (Thermo Scientific). Proteins were separated by SDS-PAGE (NuPAGE, Invitrogen), transferred to PVDF membranes (Millipore). The membranes were blocked with 5% skimmed milk, and blotted with specific antibodies. HEK-293T cells were transfected (Lipofectamine, Invitrogen) with plasmids encoding wild-type or mutated FLAG-tagged Stx11 (FLAG-Stx11) constructs, wild-type HA-tagged Munc18-2 (HA-Munc18-2, the empty vector, or combinations thereof). Twenty-four hours following transfection, the cells were lysed and the protein concentration was determined by Bradford assay (Thermo Scientific). For pull-down experiments, protein G-beads (Invitrogen) were pre-incubated with anti-FLAG mAb, washed in lysis buffer, and incubated with lysates from different FLAG-Stx11 transfected cells for 2 h at 4°C. Subsequently, FLAG-Stx11-loaded beads were washed and incubated with lysates from vector or HA-Munc18-2 transfected cells for 4 h at 4°C.

## Results

### Clinical and immunological characterization of patients with a homozygous *STX11* missense mutation

Here, we describe two infants and one 5-year-old child born to unrelated Pakistani families that presented with HLH (Table 1). Patient A and B presented with a laboratory parameters consistent with a clinical diagnosis of HLH at the Aga Khan Hospital, Karachi. Patient C also presented with a hyperinflammatory syndrome and was later referred to the Aga Khan Hospital. For patient C, it has not been possible to retrieve laboratory parameters at initial presentation.

**Table 1:** **Clinical, laboratory, and genetic findings in patients**.

	A	B	C
Ethnical origin	Pakistan	Pakistan	Pakistan
Familial disease	No	Yes	No
Parental consanguinity	Yes	Yes	Yes
Sex	Male	Male	Female
*STX11*	173T > C, Leu58Pro hmz	173T > C, Leu58Pro hmz	173T > C, Leu58Pro hmz
*STXBP2*	None detected	None detected	None detected
*UNC13D*	c.811C > T p.Pro271Ser htz	None detected	None detected
	c.2782C > T p.R928C hmz	
Age at diagnosis-HLH	2 months	5 years	48 months
Fever	Yes	Yes	nd
Splenomegaly	Yes	Yes	nd
Hepatomegaly	Yes	Yes	nd
Hb (g/L)	55	71	nd
Neutrophils (10^9^/L)	0.3	0.4	nd
Platelets (10^9^/L)	13	8	nd
Triglycerides (mmol/L)	5.1	5.5	nd
Fibrinogen (g/L)	0.28	0.16	nd
Hemophagocytosis	No	No	nd
Ferritin (μg/L)	8636	1929	nd
sCD25 (U/mL)	nd	nd	nd
NK cell activity^a^	Deficient	Deficient	Deficient
NK cell degranulation	Deficient	Deficient	Deficient
Neurological manifestations^b^	None	None	nd
Pathological CSF	nd	nd	nd
Treatment active disease	Dexa, CsA, etoposide	Dexa, CsA, etoposide	nd
Remission at 2 months	Yes	Yes	Lost to follow-up
Age at HSCT	15 months	Not done	nd
Outcome	Deceased	Deceased	nd

*^a^Defective: 10 lytic units or less*.

*^b^Reported at some point during the course of the disease; nd = no data; Dexa = dexamethasone; CsA = cyclosporine A; HSCT = hematopoietic stem cell transplantation*.

**Figure 1:**
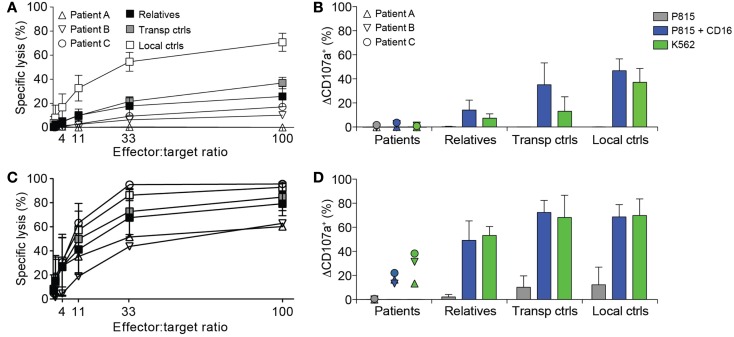
**Defective NK cell degranulation in patients with homozygous *STX11* c.173T > C mutations**. **(A–D)** PBMC were isolated from three patients with homozygous *STX11* c.173T > C, p.L58P mutations, relatives (*n* = 10, all six parents in addition to four siblings) as well as transport (*n* = 3) and local controls (*n* = 5). PBMC **(A)** freshly isolated or **(C)** stimulated with IL-2 overnight were mixed with ^51^Cr labeled K562 target cells. After 4 h, ^51^Cr-release was quantified in supernatants and specific lysis was calculated. Symbols indicate individual values for patients or mean values for relatives and controls. Error bars indicate SD. PBMC **(B)** freshly isolated or **(D)** stimulated with IL-2 overnight were mixed with target cells and antibodies, as indicated. The cells were stained with antibodies to lineage markers and CD107a. The frequency of cells expressing surface CD107a was determined by flow cytometry. Symbols indicate values for patients and bars mean for relatives and controls. Error bars indicate SD.

**Figure 2:**
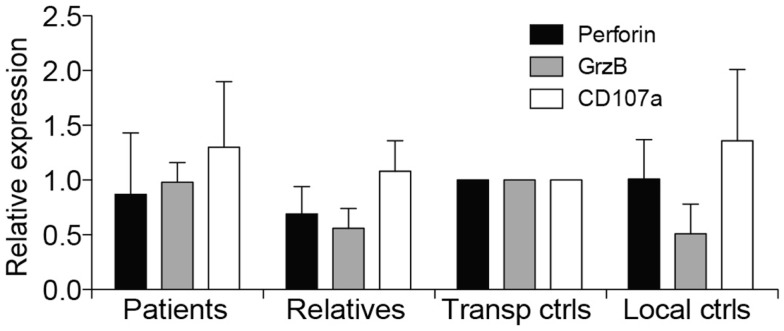
**Intracellular expression of CD107a, perforin, and granzyme B in patients with homozygous *STX11* c.173T > C mutations**. Intracellular expression of perforin, granzyme B, and CD107a was examined in patient B and C, in addition to relatives (*n* = 8, all four parents in addition to four siblings) as well as transport (*n* = 2) and local controls (*n* = 4). Expression of granule constituents was normalized relative to that of the transport control. Bars indicate the mean value, Error bars indicate SD.

Due to suspicion of FHL, NK cell cytotoxicity, degranulation, and intracellular expression of granule constituents was assessed. All patients displayed defective lysis of K562 target cells and degranulation by NK cells in response to K562 target cells or engagement of the Fc receptor CD16 (Figures 1A,B). Notably, cytotoxicity and degranulation were partially restored by IL-2 stimulation (Figures 1C,D). Moreover, expression of cytotoxic granule constituents’ perforin, granzyme B, and CD107a was normal in patient NK cells, suggesting that granule integrity was not impaired (Figure 2). On the basis of these functional and phenotypic assessments, mutations in genes required for lymphocyte exocytosis and associated with FHL were suspected.

Sequencing of the coding regions and splice-sites of *UNC13D, STX11*, and *STXBP2* revealed that all three patients were homozygous for a novel *STX11* mutation, c.173T > C (p.L58P) (Table 1). The L58P localizes to an α-helical strand of the predicted Stx11 Habc domain. The parents were heterozygous for this *STX11* mutation, but did not have any recorded history of inflammatory disease. In addition, patient A was heterozygous for a rare *UNC13D* c.811C > T (p.P271S; frequency 0.001 in a Caucasian population of 4294 individuals) variant inherited from the father and homozygous for an uncommon *UNC13D* c.2782C > T (p.R928C, frequency 0.01 in a Caucasian population of 4294 individuals) variant inherited from either parent. As no hypopigmentation was evident in the patients, *RAB27A* and *LYST* were not sequenced.

### A homozygous *STX11* missense mutation results in selective loss of SYNTAXIN-11 expression in patient NK cells

**Figure 3:**
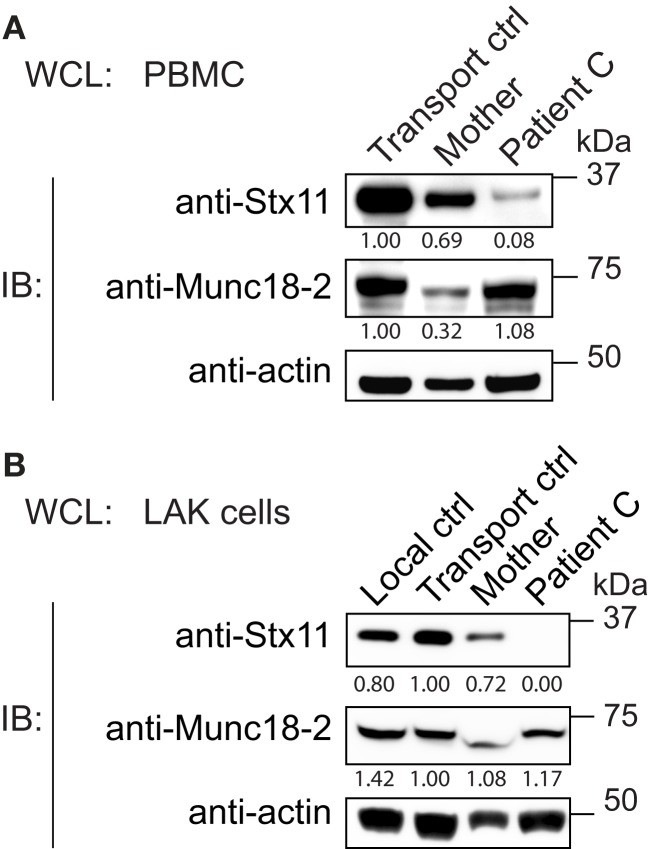
**Low expression of syntaxin-11 L58P in patient lymphocytes**. Whole cell lysates (WCL) prepared from **(A)** PBMC or **(B)** LAK cells from Stx11 L58P patient C and controls, as indicated, were analyzed by Western blotting for Stx11 and Munc18-2. β-actin was probed as a loading control. Densitometry values normalized to β-actin for each individual are indicated.

To gain insights into how the Stx11 L58P missense mutation may cause disease, we analyzed Stx11 expression in PBMCs from patient C and controls. Stx11 levels were found to be greatly reduced in the patient (Figure 3A). PBMCs from the patient’s mother displayed low Munc18-2 expression as well as slightly decreased syntaxin-11 expression. Although Munc18-2 levels were comparable between the patient and controls, the loss of Stx11 expression may reflect differences in the distribution of immune cell subsets or the inflammatory state between the patient and controls. Thus, we generated LAK cells from patient C and controls. LAK cells from the patient also displayed a selective loss of Stx11 expression, whereas Munc18-2 expression was similar to that of control LAK cells (Figure 3B). LAK cells from the patient’s mother displayed syntaxin-11 and Munc18-2 levels similar to those of control LAK cells. Thus, Stx11 L58P might either be poorly expressed or be destabilized and degraded in the patient cells.

### Syntaxin-11 R4A and L58P mutations disrupt binding to Munc18-2

**Figure 4:**
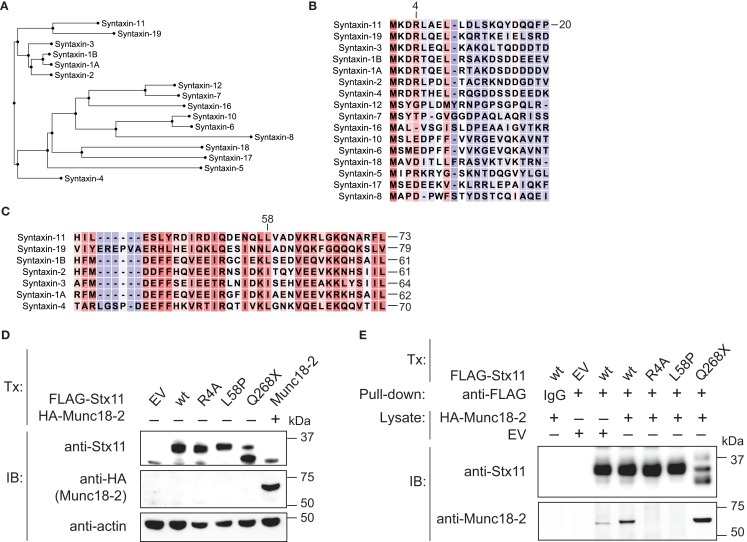
**Abrogated binding of Munc18-2 to syntaxin-11 N-terminal R4A or Habc-domain L58P mutations**. **(A)** Dendrogram comparing human syntaxins based on peptide sequence alignments of the N-termini (N-peptide and Habc domain; residues 1-167 of Stx11). **(B)** Amino acid alignments of human syntaxin **(B)** N-terminus and **(C)** Habc domain sequences. Red shade indicates a high degree of sequence homology, whereas blue shade indicates low homology. **(D)** HEK-293T cells were transfected with empty vector (EV), or constructs encoding FLAG-Stx11 wild-type (wt), R4A, L58P, Q268X, or HA-Munc18-2 wild-type. Twenty-four hours after transfection, WCL were prepared and analyzed for expression of Stx11, Munc18-2, and actin. **(E)** HEK-293T cells constructs as indicated. Twenty-four hours after transfection, WCL were prepared from Stx11 transfectants and Stx11 was immunoprecipitated to beads. The beads were washed extensively and thereafter incubated with WCL from EV or HA-Munc18-2 wt transfected HEK-293T cells. Beads were washed, proteins eluted, and analyzed by Western blotting with antibodies as indicated.

Stx11 interacts with Munc18-2 and loss of Munc18-2 expression has previously been shown to result in loss of Stx11 expression as well, suggesting a requirement for Munc18-2 in stabilization of Stx11 (9, 10). With respect to N-terminal peptide sequences, human Stx19, Stx1A, Stx1B, Stx2, Stx3, and Stx4 represent close homologs to human Stx11 (Figure 4A). Remarkably, the N-peptide as well as the sequence surrounding the Stx11 L58 residue in the Habc are highly conserved between Stx11 and Stx1 isoforms, as well as other related Stxs (Figures 4B,C). Interestingly, studies of neuronally expressed Stx1A and Munc18-1 have revealed that the N-terminal residues (N-peptide) as well as the N-terminal Habc domains of Stx1 mediate interactions with Munc18-1 (20–22). As both the N-peptide and Habc domain of Stx1A are closely conserved to those of Stx11, we evaluated whether a Stx11 R4A mutation as well as the patient-derived Stx11 L58P mutation located to the Habc domain would interfere with binding of Munc18-2. Constructs for ectopic expression of FLAG-tagged wild-type and mutant Stx11 were transfected into HEK-293T cells. In transfection experiments, both Stx11 R4A and L58P mutants were expressed at levels comparable to Stx11 wild-type (Figure 4D). Notably, in pull-down experiments using beads loaded with FLAG-tagged Stx11 wild-type and Stx11 mutants neither FLAG-tagged Stx11 R4A nor L58P mutants bound HA-tagged Munc18-2 (Figure 4E). In contrast, the C-terminal Stx11 Q268X mutation previously associated with FHL4 (14), did not display impaired binding of Munc18-2 (Figure 4E). Together, the data show that mutations in the N-peptide or Habc domain of Stx11 can disrupt interactions with Munc18-2, demonstrating a critical role for both the N-peptide and Habc domain of Stx11 in binding of Munc18-2.

## Discussion

We describe a novel autosomal recessive missense *STX11* c.173T > C (p.L58P) mutation causative of FHL4 in three children from different Pakistani kindreds. Stx11 expression was absent in NK cells from a patient homozygous for this *STX11* mutation. Consistent with previous studies, the *STX11* mutation was associated with defective degranulation by resting NK cells (14, 23). Of note, whereas only *STX11* mutations were detected in the other patients, patient A also carried *UNC13D* variants. By comparison, this patient presented at an earlier age than the patient B and C, and displayed less of a restoration of NK cell degranulation upon IL-2 stimulation. Thus, although an abrogation of NK cell degranulation is expected in Munc13-4 deficient patients, it is possible that the *UNC13D* variants contribute to the severity of disease presentation in patient A.

By sequence homology to Stx1A, the Stx11 L58P mutation is located in the first α-helix of the conserved Habc domain of Stx11 (20). Substitution to a proline residue at this position likely disrupts the conformation of the Stx11 Habc domain. Interestingly, biochemical analyses examining ectopically expressed, tagged Stx11 in a cell line revealed that the Stx11 L58P mutation disrupted interactions with tagged Munc18-2. Mutations in *STXBP2* that lead to loss of Munc18-2 expression also cause loss of Stx11 expression in lymphocytes (9, 10). Thus, conversely, it is tenable that Stx11 mutations that disrupt Munc18-2 binding might reduce Stx11 expression through a similar mechanism, explaining the low expression of mutant Stx11 in patient cells.

With respect to neuronal Stx1 binding of Munc18-1, the very N-terminal residues as well as the Habc domain of Stx1 mediate a binary interaction with Munc18-1 (20–22). Our data suggest that Stx11 binding to Munc18-2 has similar molecular requirements as either mutation of the conserved Stx11 N-peptide (R4A) or of the Habc domain (L58P) abrogated Munc18-2 binding. These observations are supported by recent publications demonstrating that mutations in the hydrophobic pocket of Munc18-2, which can bind the N-peptide of Stx11, abrogate Stx11 binding and mast cell degranulation (24). During preparation of this manuscript, the Munc18-2 crystal structure was reported (25). The crystal structure, as well as studies of how Munc18-2 mutations associated with FHL5 impact Stx11 binding, similarly suggest a requirement for the N-peptide and Habc domains of Stx11 for binding to Munc18-2 (25). With respect to neuronal exocytosis, it has recently been shown that mutations in the Habc domain of Stx1 abrogate interactions with Munc18-1, which usually keep Stx1 in a closed conformation, leading to reduced Mun18-1 expression (26). In contrast, mutations of the Stx1 N-peptide more specifically interfere with vesicle fusion (26). It will be interesting to further determine how the N-terminus and Habc domain of Stx11 regulate Stx11 conformation, trafficking, and granule exocytosis.

Stx11 has been shown to interact with additional proteins involved in vesicle exocytosis, including SNAP-23, VAMP2, and Vti1b (16, 17). In addition, the priming factor Munc13-4 has been shown to interact with different Stxs (27). These proteins interact with the Stx11 C-terminal SNARE domain, with preferential binding to the open conformation of Stx11. Speculatively, although other more direct mechanisms for degradation of Stx11 due to protein misfolding also may explain low Stx11 expression, disruption of the Stx11 – Munc18-2 interaction in patient NK cells may lead to Stx11 degradation based on a mechanism dependent on such facilitators and regulators of vesicle exocytosis. Thus, it is of interest to perform a more comprehensive screen of how different Stx11 mutations impact interactions with other proteins implicated in facilitating and regulating granule exocytosis.

In conclusion, we demonstrate that both the N-terminus and Habc domain of Stx11 are involved in binding to Munc18-2. In the patients homozygous for a Stx11 L58P mutation, it is quite possible that the abrogated interaction between Stx11 and Munc18-2 leads to destabilization of Stx11 expression. Further studies of Stx11 mutants may provide insights into mechanisms, specificity, and redundancy governing SNARE complex formation for lytic granule exocytosis by cytotoxic lymphocytes.

## Author Contributions

Martha-Lena Müller designed research, performed biochemical experiments, analyzed and interpreted data, and drafted the manuscript; Samuel C. C. Chiang designed research, performed functional evaluations of lymphocytes, analyzed and interpreted data, and drafted the manuscript; Marie Meeths designed research, performed targeted sequencing of FHL genes, and drafted the manuscript; Bianca Tesi designed research and performed targeted sequencing of FHL genes; Miriam Entesarian, Daniel Nilsson, and Stephanie M. Wood designed experiments and interpreted data; Magnus Nordenskjöld and Jan-Inge Henter designed research and interpreted data; Ahmed Naqvi identified and cared for patients, collected clinical data, and drafted the manuscript; Yenan T. Bryceson designed research, interpreted data, and drafted the manuscript.

## Conflict of Interest Statement

The authors declare that the research was conducted in the absence of any commercial or financial relationships that could be construed as a potential conflict of interest.
